# The management of sexually transmitted infections: a scoping survey in primary care

**DOI:** 10.3399/bjgpopen18X101639

**Published:** 2019-04-17

**Authors:** Jayshree Dave, John Paul, Julie Johnson, Jane Hutchinson, Glenn Phiri, Asha Dave, Neville Verlander, David Carrington

**Affiliations:** 1 Consultant Microbiologist, Public Health Laboratory London, National Infection Service, Public Health England, London, UK; 2 Honorary Clinical Professor, Brighton and Sussex Medical School, Department of Global Health and Infection, University of Sussex, Falmer, Brighton, UK; 3 Regional Head of Operations for London & South East England and the NMRS-S, National Infection Service, Public Health England, London, UK; 4 Primary Care Lead in Sexual Health, All East Integrated Sexual Health Services, Barts Health NHS Trust, London, UK; 5 Business Officer, Health Care Consulting, North East London Commissioning Support Unit, London, UK; 6 Medical Student, UCL Medical School, University College London Medical School, London, UK; 7 Statistician, Statistics, Modelling and Economics Department, National Infection Service – Data and Analytical Sciences, Public Health England, London, UK; 8 Consultant Virologist, Department of Medical Microbiology, St George's University Hospitals NHS Foundation Trust, London, UK

**Keywords:** General practitioners, Sexually transmitted infections, Diagnosis, Antibiotics, Treatment, General practice

## Abstract

**Background:**

National guidelines for sexually transmitted infections (STIs) in primary care exists but their management is uncertain.

**Aim:**

To assess the management of STIs against national standards in primary care.

**Design & setting:**

A questionnaire based study in London and Brighton. The survey was conducted in 2015 following reorganisation of sexual health services in England.

**Method:**

Questionnaires were sent to GPs in London and Brighton about testing for STIs, treatment for gonorrhoea, specialist advice, and referral services.

**Results:**

Of 119 GPs who responded, most expressed confidence in treating chlamydia (*n* = 105/119, 88%), trichomonas (*n* = 81/119, 68%), and herpes (*n* = 82/119, 69%) but not gonorrhoea (*n* = 32/119, 27%). Most referred cases of syphilis (*n* = 92/119, 77%) and genital warts (83/119, 70%) to genito-urinary medicine (GUM) as per guidance. Most GPs tested for gonorrhoea on patient request (*n* = 95/119, 80%), in tandem with chlamydia screening (*n* = 89/119, 75%), because of high risk status (*n* = 85/119, 71%) and genital symptoms (*n* = 108/119, 91%). Some GPs (*n* = 22/119, 18%) sampled urine for culture, 53/119 (45%) provided high vaginal swabs (HVS), and 28/119 (24%) provided self-taken vulvovaginal swabs (STVVS) for culture. These samples are not appropriate for gonococcal culture and not processed in the laboratory. Urethral swabs for men and endocervical swabs (ECS) are recommended for gonococcus culture. Over half (*n* = 60/102, 59%) of GPs did not treat gonorrhoea but some prescribed cefixime, ciprofloxacin, or azithromycin. Eighty-seven per cent (*n* = 104/119) sought advice from GUM, and 83/103 (81%) referred gonorrhoea nucleic acid amplification test (NAAT)-positive patients.

**Conclusion:**

There is scope for improvement of STIs management in primary care to ensure that patients are optimally investigated, treated, and referred.

## How this fits in

This scoping survey revealed that GPs were not fully compliant with national guidance for the management of STIs in primary care. Most GPs expressed confidence in treating chlamydia, but lacked confidence in treating genital gonorrhoea. Urethral swabs (for men) and ECS (for women) are recommended for gonococcus culture, but some GPs sent urine for culture and provided high vaginal swabs and STVVS for culture, samples which would not be processed in the laboratory. Many GPs refer patients for treatment of gonorrhoea but a few inappropriately prescribed cefixime, ciprofloxacin, and azithromycin as single agents.

## Introduction

Patients with STIs are often managed initially by GPs. Sexual health provision in England has undergone major changes following the implementation of the Health and Social Care Act 2012, resulting in shared commissioning between NHS England, local authorities, and clinical commissioning groups,^1^ and commissioning of internet-accessible sexual health services.^2^ London and Brighton have the highest and second highest reported rates of sexually transmitted infections in England respectively.^3^ National guidance exists for STIs management in primary care.^4^ Also, national standards exist for providers of sexual health services.^5^ As many patients still access services provided by GPs, it is important to understand how they manage STIs. Wetten *et al* ^6^ demonstrated the continued use by some GPs of antimicrobials no longer recommended for gonorrhoea. It is also pertinent to assess how primary care clinicians screen patients for STIs, and utilise laboratory and referral services. The objective of this survey was to understand the management of STIs in primary care in London and Brighton, two cities with high rates of STIs. The questionnaire concentrated upon six key areas: confidence in treating STIs, indications for sampling, sample types, gonorrhoea treatment, accessing clinical advice and referral pathways for gonorrhoea.

## Method

A questionnaire was developed by collaboration with microbiologists, GUM physicians, a primary care facilitator, and GPs to investigate the management of STIs in primary care, focusing on confidence in the diagnosis of STIs, indications for sampling, sample types, treatment, accessing clinical advice and GUM referral (available from the author on request). The national guidance for sexually transmitted infections, developed by the Royal College of General Practitioners and British Association for Sexual Health and HIV (BASHH), was used as the standard^4^ for developing the questionnaire. GPs in London and Brighton were targeted, cities which have high rates of STIs.^3^ All 1406 general practices in London and 329 practices in the Brighton area were invited to participate in this survey by email correspondence. The letter was distributed via an up-to-date practice directory held by the pathology departments in Barts Health NHS Trust London and Royal Sussex County Hospital,Brighton. The message explained that the questionnaire required a response from GPs on their management of STIs with emphasis on *Neisseria gonorrhoeae*. The email stated that individuals presenting at risk of an STI, with or without symptoms, are increasingly seen in primary care. The survey examined the care of patients with STIs in collaboration with the local GUM service. It was stated that BASHH has developed guidance on STIs in primary care, and standards have been developed to support all providers of sexual health services in achieving safe, high quality services for the management of STIs. An electronic link to the ‘select survey’ tool provided access to the questionnaire, which comprised 16 questions. Fifteen questions were closed-ended and one was open-ended. Some of the questions had a tabular format, with multiple rows and columns which required a tick box reply. Five of the closed-ended questions also had one stem for open-ended replies. Each question required a response before the individual could respond to the next question. Potential response bias was addressed by improving the clarity and length of the questions, by avoiding leading questions, using simple and precise language, and by ensuring the questions considered the target audience. Selection bias was avoided by sending the questionnaire to all the practices in London and Brighton. As achieving a 10%–15% response rate was anticipated to be challenging, all GPs in London and Brighton were targeted. Potential confounding was determined by testing the questionnaire with GPs and developing the questionnaire using key stakeholders from microbiology, GUM, and the primary care facilitator and by avoiding specific requested values and using categories. The survey would take 5 minutes to complete and the responses were anonymised. The select survey tool is widely used within Public Health England and the questionnaire was available for 3 months for completion. The questionnaire was sent to general practices in London five times and in Brighton twice in 2015–2016.

## Results

Of 164 GPs who accessed the questionnaire, a maximum of 119 responded adequately to survey questions. As seen in the tables below, some responders did not address all the questions and some of the sections within each question were incomplete.

As shown in Table 1, confidence for treating gonorrhoea varied by STIs, with a larger proportion of GPs being confident for treating chlamydia , trichomonas, and genital herpes compared to genital and extragenital gonorrhoea, genital warts, and syphilis. This pattern was reflected in the number of responders seeking specialist advice and referral to GUM.

**Table 1.** Responder answers to the question ‘Do you feel confident treating the following STIs? Or would you prefer to seek advice or refer for treatment?’ (*n* = 119)

Type of STIs	I am confident treating, % (*n*)	I would ask for advice, % (*n*)	I would refer to the GUM clinic, % (*n*)	Other (give details below), % (*n*)	Response total, *n*
Chlamydia	83 (105)	3 (4)	11 (14)	2 (3)	126
Genital gonorrhoea	25 (32)	22 (28)	49 (63)	4 (5)	128
Extragenital gonorrhoea	4 (5)	24 (30)	69 (86)	2 (3)	124
Trichomonas vaginalis	64 (81)	14 (17)	21 (26)	2 (2)	126
Genital warts	22 (28)	9 (11)	66 (83)	3 (4)	126
Genital herpes	66 (82)	11 (14)	21 (26)	2 (2)	124
Syphilis	4 (5)	21 (27)	73 (92)	2 (2)	126

Proportions may not add up to 100 due to rounding of numbers

GUM = genito-urinary medicine. STI = sexually transmitted infection.

The majority of GPs tested for gonococcus upon patient request, as part of chlamydia screening (*n* = 89/119, 75%), due to high risk status or presence of genital symptoms but fewer tested for extragenital symptoms (Table 2).

**Table 2.** Responders’ indicators for testing for gonococcus in general practice

What are the usual indicators for gonococcus testing in your practice?	*n* (%)
Patient requests the test	95 (80)
Part of chlamydia screening	89 (75)
High-risk patient	85 (71)
Symptomatic, genital symptoms	108 (91)
Symptomatic, extragenital symptoms	42 (35)
Other, please specify	7 (6)

Eighteen GPs (15%) reported that they would take urethral swabs and 43/119 (35%) would submit urine for NAAT (Table 3). Fifty-seven (48%) reported that they would not routinely test for gonorrhoea (Table 3). However, 44/119 (37%) GPs submit ECS, 25/119 (21%) HVS, and 43/119 (36%) STVVS (Table 3).

**Table 3.** Responder answers to the question ‘What sample types do you routinely take for gonococcus testing?’ (*n* = 119)

Sample type	Specimen for MC&S for gonorrhoea, % (*n*)	Specimen for NAATs for gonorrhoea, % (*n*)	Samples not routinely taken for gonorrhoea, % (*n*)	Responses total, *n*
Urethral swab	22 (28)	14 (18)	63 (79)	125
High vaginal swab	42% (53)	20 (25)	38 (47)	125
Self-taken vulvovaginal swab	23 (28)	35 (43)	42 (51)	122
Endocervical swab	38 (47)	36 (44)	26 (32)	123
Throat swab	9 (11)	8 (10)	82 (98)	119
Rectal swab	10 (12)	11 (13)	79 (95)	120
First pass urine	18 (22)	35 (43)	47 (57)	122

Proportions may not add up to 100 due to rounding of numbers. NAAT = nucleic acid amplification test. MC&S = microscopy, culture and sensitivity

Only 102 responded to the question on whether they prescribed antibiotic treatment for gonorrhoea infection. Many GPs (*n* = 60, 59%) would not prescribe treatment. When asked about the antibiotic selection for treating gonorrhoea, there were 23/106 conditional responses and choices varied from cefixime with or without azithromycin, ceftriaxone with or without azithromycin, ciprofloxacin, ofloxacin, azithromycin, seeking advice from GUM, or looking at guidelines.

There were 14 respondents who did not complete the questionnaire and only responded to the first four questions, and one individual completed seven questions. The numbers were too small for a meaningful comparison between the two groups.

Seventy-six of the 103 (74%) reported a process for ensuring that partners of gonorrhoea-positive patients received treatment.

**Figure 1.**
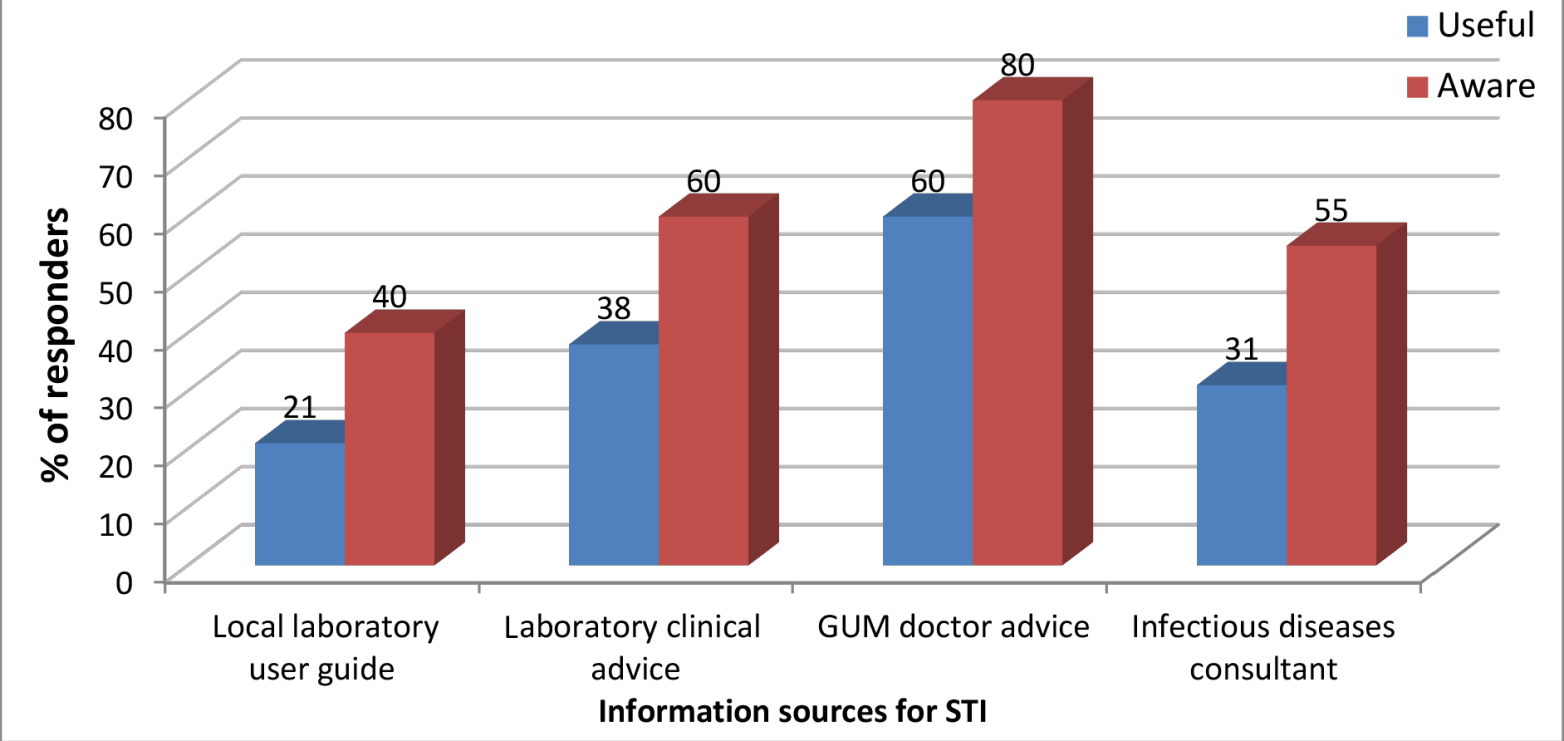
Responses to the question ‘Are you aware of any of the following sources of information on sexually transmitted infections and do you find them useful?’ GUM = genito-urinary medicine. STI = sexually transmitted infection.

Most GPs obtained information on STIs (Figure 1) and clinical advice (Table 4) from the GUM service.

**Table 4.** Results regarding access to clinical advice, management of patients with gonococcal positive NAATs, and referral pathway for GUM

**When seeking advice on STI, who would you contact?**	***n* (%)**
GUM	104 (87)
Primary care colleague	30 (25)
Primary care facilitator	1 (1)
Ask a colleague	17 (14)
BNF	39 (33)
Phone microbiologist	33 (28)
Other, please specify	4 (3)
Total responses, *n*	119
**What form of real time clinical advice on sexual health/STI would you find most helpful?**
Online electronic messaging service to lab consultants e.g. kinesis, email	45 (42)
Advice embedded within electronic requesting (e.g. sample or test choice), or reporting (e.g. antibiotic choice) systems	88 (83)
Information leaflet on the management of gonorrhoea	31 (29)
Clinical advice by telephone on a positive result/confirmation	66 (62)
Other, please specify	7 (7)
Total responses, *n*	106
**With reference to the management of patients with suspected gonorrhoea (ie, they have positive molecular NAAT test) do you … ?**
Refer all patients	83 (81)
Refer only complicated cases of infection to a GUM clinic	16 (16)
Treat all patients at surgery	4 (4)
Total responses, *n*	103

BNF = British National Formulary. GUM = genito-urinary medicine. HIV= human immunodeficiency virus. NAAT = nucleic acid amplification test. STI = sexually transmitted infection.

## Discussion

### Summary

This general practice-based scoping survey in London and Brighton provides information on confidence in treating STIs, indications for sampling, sample types, treatment for gonorrhoea, and accessing clinical advice and referral pathways for STIs diagnosed in the community. From the 1735 GP practices contacted, 119 GPs responded to the questionnaire. There was marked heterogeneity in confidence and knowledge in the management of STIs and better use of the national guidance would improve the management of these infections in the community. As screening for chlamydia is encouraged in the community,^7^ confidence in managing patients with chlamydia was found to be high. There was less confidence in managing gonorrhoea; a large number of GPs would refer cases of gonorrhoea, especially extragenital gonorrhoea, to GUM, which is in line with national guidance. Likewise, cases of syphilis and genital warts would be referred to GUM, but few would refer trichomonas to GUM, perhaps as treatment is considered straightforward with metronidazole. , GUM referral is, however, preferable to enable the screening of coexisting STIs. There was confusion regarding the sample types required for molecular diagnosis and those required for culture of gonorrhoea. ECS (recommended for gonococcal culture^8,9^ were still regarded a useful sample type for gonococcal NAAT testing (37%) when STVVS would suffice. Although urine is suitable in men for gonococcal NAATs,^8^ this was not understood by over half of the responders, and some GPs continue to send urine for gonococcal culture, which is not recommended.^9^ A large number of GPs opted not to treat gonorrhoea in general practice; if they did, cefixime, ciprofloxacin, or azithromycin were considered for treatment, which is not in line with the primary care national guidance.^4^

Advice embedded within electronic requesting was popular amongst responders and access to antibiotic treatment guidance with regular reviews would benefit antimicrobial stewardship within the community.

There was good evidence that gonococcal NAAT-positive patients and their contacts would be referred to GUM in line with national guidance.^4^

### Strengths and limitations

A strength of this scoping exercise was the use of national guidance as a standard developed for STIs in the evaluation of these findings. The questionnaire was comprehensive, with provision for comments, and covered key areas of management of these patients in primary care.

However, this scoping exercise highlighted several limitations. Although designed for GPs, participant anonymity means that other practice staff might have completed the questionnaire. This may explain discrepancies in the numbers of responders for different questions. A follow-up study should focus on key questions. Another limitation was the inability to differentiate between responses from Brighton and London, which limited analysis.

The national guidance for STIs management in primary care was not provided in the mailshots and some participants might not have been aware of it. Some GPs might have presumed the BASHH guidance in the mailshot to be secondary care guidance, and this might have affected responses to questions. Provision of the guidance document might have improved the completeness of the survey and the response rate. Question clarity might have been improved by substituting the word ‘testing’ for ‘screening’, and by providing a definition for ‘high risk’. It may be that some GPs test and treat some patients in the community and then refer to GUM for further follow up, but this was not captured in this study.

Despite repeated attempts to encourage participation only 119 responses were received from the more than 1000 practices in London and Brighton. Unfortunately, this is not surprising given the many competing demands on time in general practice. The results provided should thus be interpreted as merely applying to the practices from which the information was collected. Differences in the number of emails to GPs in the two areas reflected local communication practices. This represented a potential source of bias in interpretation of the results.

### Comparison with existing literature

From 1735 general practices in London and Brighton, 119 responses were obtained. This may be because some GPs may not have seen or been aware of the questionnaire as it was sent electronically as a link. It is difficult to give an indication of how representative the sampling frame was to provide a definite understanding of the management of STIs. While the findings may not reliably represent practice and knowledge in the wider community, some clear learning points emerged regarding the management of STIs.

GPs reported varying levels of confidence and knowledge in managing STIs in general practice. Whilst levels of confidence were high for chlamydia, lower levels of confidence were found for gonorrhoea. A large number of GPs were confident about treating genital herpes and this may reflect the more frequent requirement to treat recurrent rather than primary genital herpes infection. Most GPs were confident about management of patients with trichomonas infection. However, the increasing availability of molecular tests for trichomonas, the poor performance characteristics of trichomonas culture and its main utility in patients from a high-risk population (for example, Black African),^10^ and the investigation of coexisting STIs would suggest that GUM referral would be beneficial to patients. Most GPs would refer syphilis and genital warts cases to GUM, as advised by the guidance,^4^ reflecting the complexity in the management of these cases.

There was some evidence of confusion regarding the testing of patients for gonorrhoea and their treatment and referral. National guidance recommends NAAT to investigate suspected cases of gonorrhoea. Despite evidence that STVVS are non-inferior to ECS and high vaginal swabs for NAAT,^8,11^ 37% of GPs submitted ECS, possibly because this is the recommended sample type for culture.^8,10^ National guidance^4^ recommends culture of NAAT-positive patients in order to determine antibiotic susceptibilities. For women, only 45% of GPs would submit ECS for culture, possibly because of confusion with guidance for NAAT samples. In men, urethral swabs are recommended for gonococcal culture but, surprisingly, 18% of GPs stated they would submit urine samples, possibly because urine is a suitable sample type for NAAT. Gonococcus is a fragile organism and the laboratory recovery rate^12^ is poor with increased transport time. GPs may not be aware that successful culture is very dependent on getting the sample to the laboratory quickly.^12^

A considerable number of GPs said their practice would not prescribe treatment for gonorrhoea in accordance with national guidance,^4^ which advises referral to GUM unless this is difficult to arrange. Some replies complied with the use of recommended antibiotics for treating gonorrhoea, but a few GPs would use cefixime, ciprofloxacin, and azithromycin, suggesting that information about antibiotic treatment with regular updates may improve prescribing particularly as the majority of the GPs wanted advice embedded within electronic requesting. A recent study found that only 1.7% (*n* = 34/1956) of all gonorrhoea cases were diagnosed in primary care and there was high correct pathway management.^13^

Changes in STI guidance (for example, BASHH chlamydia treatment guidelines)^14^ present primary care services with the challenge of keeping up to date. This may cause variation in treatment guidance followed by different local health institutions.

Reports of the emergence of multi-drug resistance in *N* *eisseria gonorrhoeae* ^15^ emphasises the importance of gonococcal treatment guidance with regular reviews thus improving antimicrobial stewardship in the community.

Reassuringly, most GPs would not consider management in the community of patients and their contacts with gonorrhoea who were NAAT-positive, and would refer to GUM, in accordance with national guidance^4^ and helping to reduce further selection pressure for antimicrobial resistance.

### Implications for research and practice

In a changing landscape of provision of care for STIs, these results suggest areas for targeted education and training in the management of STIs among GPs. Future work could involve a different study design with an interview process involving key questions, improved by addressing the limitations discussed above, with a limited number of GPs. This would provide meaningful data for analysis and potentially improve the management of these patients.
